# The future of date palm cultivation in the Lower Jordan Valley of the West Bank

**DOI:** 10.1007/s13201-018-0746-2

**Published:** 2018-06-29

**Authors:** B. G. J. S. Sonneveld, A. Marei, M. D. Merbis, A. Alfarra

**Affiliations:** 1 Amsterdam Centre for World Food Studies, Vrije Universiteit Amsterdam, Amsterdam, The Netherlands; 2 Department of Earth and Environmental Sciences, Faculty of Science and Technology, Al Quds University, Jerusalem, Palestine; 3 Land and Water Division, Food and Agriculture Organization of the United Nations, Rome, Italy

**Keywords:** Date palm, Irrigation, West Bank, Salinity, Cost benefit analysis

## Abstract

High water consumption and specific soil requirements warrant a long-term planning for date palm cultivation. Hence, this study presents a detailed procedure to calculate water and land balances that assess the suitability for date palm cultivation in three districts of the West Bank. It applies crop response functions to relate spatially explicit land suitability and salinity levels to net revenues. Furthermore, it compares net present values and benefit–cost ratios under various discount rates and salinity levels to assess economic feasibility. Date palm cultivation in Jericho-Al Ghoor is economically achievable, but additional land amendments are required for expansion in Nablus and Tubas districts. Prevailing average salinity levels have minor negative influence on future date palm developments.

## Introduction

The agricultural sector is an important contributor to the Palestinian economy. It produces 11–20% of GDP, accounts for 25% of total exports and is the third largest employer engaging 15% of the total work force (Alatawneh 2013). Though its relative importance has declined since 1967 (UNCTAD 2015), the agricultural sector remains a key growth component for the Palestinian economy and became stronger since 2003 (Kousa 2003). Substantial growth was recorded in 2011 when the sector contributed 5.5% of the GDP and 15% of total employment (Palestinian Central Bureau of Statistics 2016). However, UNCTAD (2015) reported that of the 292,000 workers employed in agriculture, about 94% are unpaid family members, while paid workers account for less than 6% of the total of agricultural employees (Palestinian Monetary Authority, Palestinian Central Bureau of Statistics and Economic Policy Research Institute 2012). In addition, more than a quarter or 1.6 million Palestinian households are significantly or moderately food insecure, which is strongly related to the high prevalence of unemployment, with women and youth as most affected groups (UNCTAD 2014). Indeed, the creation of employment and income-generating activities is one of the main challenges facing the Palestinian authorities and potential donors in the coming years. Large-scale expansion of the agricultural sector is one of the options to achieve this, if restrictions on land and water resources could be relaxed. PMNEARI (2011) estimates that the Palestinian agricultural sector could contribute to more than 25% of GDP when targeted investment in infrastructure and expansion of irrigated areas is made, especially in Area C (under full Israeli control) and the Jordan Valley.

**Fig. 1:**
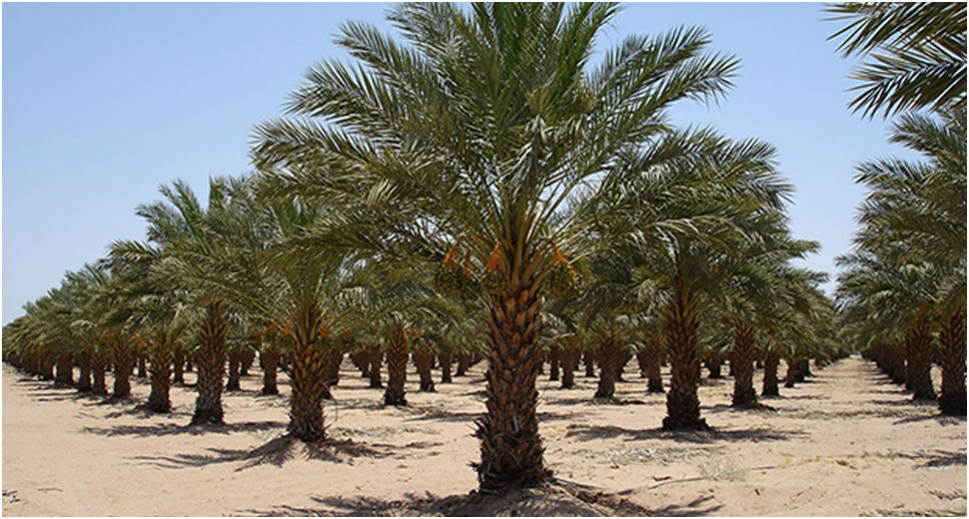
Date palm plantation *Source*: islam21c.com

It is commonly agreed that investments in the agricultural sector should focus on the development of high-value commodity chains and stimulate entrepreneurship, while generating more income for all participants involved. In line with this argumentation, opportunities that are offered by the Majhool date palm (Fig. 1) are a particular case in point. The Majhool date offers an excellent marketing position, witness the study of Zaid (2010), with Switzerland, Austria, and Belgium as leading import markets, and good prospects in many other European countries (FAO 2003). India was listed as the largest importer of Majhool date in quantity terms, followed by Malaysia and Indonesia (Sirisena et al. 2015). Moreover, a study commissioned by FAO (2003) shows that Majhool dates attract major interest in the UK and France; statistics from the US Economic Research Service (ERS 2016) show increasing import volumes for date palm fruits as well. Experts (www.freshplaza.com) estimate that trading volumes of Majhool dates are still on the low side and that world production could increase even without affecting prices. El-Jafari and Lafi (2004) compared prices of different date fruits (Majhool, Deglet Noor, Barhi, Hayani and Iraqi varieties) and found that the Majhool fruit had the highest market value (up to 3.5 USD/kg). In short, there seems to be ample marketing opportunities for the Majhool dates. Furthermore, Majhool date palm is cultivated in arid areas like North Africa, South California, Namibia, South Africa (Abu-Qaoud 2015) and Australia (Sirisena et al. 2015), where climatic conditions are similar to those prevailing in the West Bank (Zaid and de Wet 2002). Cultivation of the Majhool date palm was introduced to the West Bank by Israeli farmers in 1989, who started with a few hundred trees gradually increasing to about 250,000 in 2014. Date fruit production reached 5000 tons in 2015 and became an important export commodity for the Palestinian economy. Date palm cultivation also accommodates a social fabric since labour requirements are high (170 working days/year/hectare (FAO 2002). Labour requirements are even more intensive during pollination and harvesting/packing periods, but somewhat lower for other operations (bunch thinning, pulling down and tying, covering bunches, irrigation, pruning, fertilization).

Hence, economic profitability and employment prospects could justify the expansion of the Majhool date palm, facilitated by favourable climatic conditions and low sensitivity to salinity of irrigation water. Even under strong demand in international markets, the date fruits can be sold at remunerative prices. Furthermore, date exports may navigate rather easily through the complexities of Israeli-governed export regulations and barriers (Marei 2018). This favourable position motivates the main objective of this study: the evaluation of the biophysical and economic feasibility of expanding date palm cultivation on the West Bank.^1^

Traditionally, feasibility of agricultural projects is being assessed in two separate steps. On the one hand, soil and water resource suitability for specific crop requirements is assessed through a land evaluation procedure. On the other hand, the selected sites are subject to a cost–benefit analysis that determines economic feasibility under various discount rates. The scientific contribution of this study is to present an integrated approach that evaluates the possibilities for date palm expansion in a cost–benefit analysis that both allows for heterogeneous land quality and accounts for the prevailing geographical diversity of land qualities and water salinity levels.

To achieve this aim, we introduce crop response functions that calculate economic revenues of crop production, accounting for land suitability, water availability and water salinity levels. The response functions are expressed in net revenues: the difference between gross revenues (crop areas multiplied by yields and farm gate prices) and costs of production (irrigation water, fertilizer and pesticides, labour input). Stepwise our approach is as follows. First, we make an inventory of water balances and water quality, including the presence of underutilized water resources. Second, we categorize the land suitability by district in classes, based on prevailing land and soil attributes that favour the conditions of date palm cultivation. Third, we prepare a land balance indicating by land suitability class the available non-occupied area in selected districts. Fourth, we apply water response functions to calculate net revenues for the newly cultivated areas, while accounting for land suitability and changes in salinity levels. Finally, we integrate the information from the crop response functions in a cost–benefit analysis that presents the net present value and cost–benefit ratio over the economic life span of the date palm covering a full replanting scheme, under various discount rates and salinity levels.

**Fig. 2:**
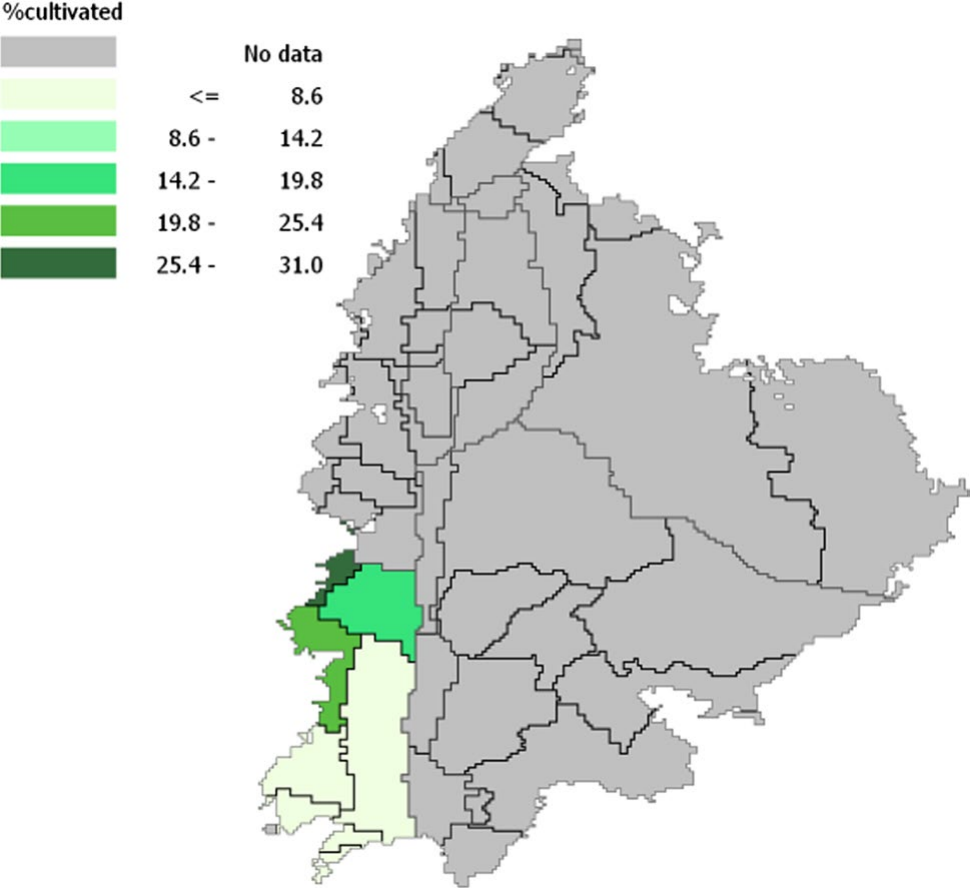
Location of study area within the JRB

For our study, we restrict to the West Bank area that is covered by the Jordan River Basin (JRB) and shares seven districts with the JRB, hereafter referred to as the JRB districts (Fig. 2). The hydrology of the JRB has been studied extensively, yielding a well-documented and detailed database^2^ that will be used for the evaluation of the date palm cultivation. The restriction to the JRB is mild, since 99% of the date palm is cultivated in just three of the seven JRB districts, namely Jericho, Tubas and Nablus.

The paper is organized as follows. For each of the JRB districts, we prepare water balances by layer and land balances based on the soil and land suitability assessment for date palm cultivation. Water response functions are introduced that relate water availability, water quality and land suitability to attainable yields of the date palm. A detailed inventory of costs and benefits during the life cycle of a date palm is presented. Results of water and land balances are presented as well as the results of the cost–benefit analysis under various levels of salinity and discount rates using the water response functions. The final section concludes.

## Data and methodology

This section discusses the water and land balances in the JRB districts and specifies the functional form of the crop response function and the cost–benefit analysis. This section presents the theoretical approach; results of implementation are discussed in the next section.

### Water balance

One of the most important components that govern a possible expansion of the date palm cultivation is the availability and quality of the water, which primarily depend on climatic conditions. With respect to date palm cultivation, we note that (a) temperature should not be less than 10 °C, (b) no rainfall should occur during the pollination period March–April, (c) no rainfall should occur during the harvesting period in August and September, and (d) no frost period is allowed. These climatic requirements are met in the Lower Jordan Valley. Additional water resources in the West Bank can be derived from groundwater and rainfall harvesting. Surface water is still unutilized due to Israeli restrictions on dam construction and access to the Jordan River, but flows from surface layer, root zone and aquifer can be accessed. Detailed information on the inflow and outflow for each of these elements can be found in van Veen et al. (2016; section 2.4 and Annex A). We discuss the use of each of the sources for the date palm cultivation here.

#### Land surface layer

The water balance in the land surface layer is characterized by inflows from rainfall corrected by the immediate evaporation and outflows from runoff to rivers, percolation to the root zone and transfer of water to anthropogenic activities through water harvesting.

#### Root zone

The root zone receives water through irrigation and leakages and percolation from the surface layer. Outflows are represented by evaporation (through crops and non-agricultural vegetation/land use), leakages and percolation to the aquifer zone.

#### Aquifer zone

The aquifer zone receives water from percolation as well as leakages from irrigation from the root zone, representing total gross recharge. Groundwater pumping and natural springs are used for anthropogenic activities, while springs also transfer water to rivers. Lateral flows between different aquifers and within aquifers spanning different districts and/or extending beyond the boundaries of the JRB lead to inflow and outflows within (Table 1).

**Table 1:** Districts sharing the JRB catchment: area and population distribution

District	Share district area in JRB	Area in km^2^ JRB	Population Palestinians	Population settlers
Jericho and Al Ghoor	0.995	724	45.206	7.307
Bethlehem	0.059	36	19.07	0.602
Jenin	0.132	75	33.153	
Jerusalem	0.493	159	90.504	86.84
Nablus	0.398	242	144.778	1.447
Ramallah-Al Bireh	0.358	283	125.425	13.873
Tubas	1.000	358	63.003	3.81
		1877	521.139	113.879

### Land balance

The land balance represents the availability and suitability of land for date palm cultivation. We start with a land assessment where suitability criteria for date palm cultivation are based on Salah et al. (2016), Darwish and Kawy (2014), Shalaby et al. (2006) and expert knowledge (Marei 2016). Land and soil data were extracted from the Harmonized World Soil database (FAO/IIASA/ISRIC/ISS-CAS/JRC 2012) and the Global 30 Arc-Second Elevation Map from the USGS data repository. Table 2 shows soil attribute classes that were considered in the land suitability assessment; suitability classes indicate from first (1) to last (5) the increasing restrictions for date palm cultivation.

**Table 2:** Soil attributes and land suitability classes for date palm cultivation

Land/soil attribute	Attribute classes
Slope class (%)	1 = 0–2; 2 = 2–5; 3 = 5–8; 4 = 8–15; 5 = 15–30; 6 = >30
Reference soil depth (cm)	1 = 100, 2 = 30, 3 = 10
Water storage capacity (mm/m)	1 = 150; 2 = 125; 3 = 100; 4 = 75; 5 = 50; 6 = 15; 7 = 0
Topsoil gravel (%)	1 = t_gravel ≤ 10; 2 = 10 < t_gravel ≤ 20; 3 = tgra_suit > 20
Topsoil organic matter (%)	1 = t_oc < 0.6; 2 = ≤0.6 t_oc < 1.2; 3 t_oc = >1.2
Topsoil calcium carbonate (%)	1 = t_caco3 ≤ 2; 2 = 2 < t_caco3 ≤ 15; 3 = t_caco3 > 15
Subsoil gypsum (%)	1 = s_caso4 ≤ 5; 2 = 5 < s_caso4 ≤ 25; 3 = s_caso4 > 25
Topsoil electronic conductivity (dS/m)	1 = t_ece ≤ 4; 2 = t_ece ≤ 4

- Slope classes were derived from the Global 30 Arc-Second Elevation Map using the ILWIS slope algorithm to estimate the slope in percentages.
- Reference depth of the soil unit is set at 100 cm, except for Rendzinas and Rankers and Leptosols of (30 cm), and Lithosols and Lithic Leptosols (10 cm).
- Available water storage capacity (AWC) in mm/m of the soil unit is subdivided in seven classes^3^: 1 = >150; 2 = 125–150; 3 = 100–125; 4 = 75–100; 5 = 50–75; 6 = 15–50; 7 = 0–15.
- Topsoil gravel indicates the volume percentage of gravel in the topsoil. Gravel stands for the percentage of materials in a soil that are larger than 2 mm.
- Percentage organic carbon in topsoil. Organic carbon is together with pH, the best simple indicator of the health status of the soil. Moderate to high amounts of organic carbon are associated with fertile soils with a good structure.
- Calcium carbonate (lime) content in topsoil is the active ingredient in agricultural lime. Low levels of calcium carbonate enhance soil structure and are generally beneficial for crop production; higher concentrations may induce iron deficiency and when cemented limit the water storage capacity of soils.
- Calcium sulphate (gypsum) content in topsoil. Up to 2% favours plant growth, between 2 and 25% has little or no adverse effect, but more than 25% can cause substantial reduction in yields.
- Electrical conductivity of topsoil. Crops vary considerably in their resistance and response to salt in soils. Some crops will suffer at values as little as 2 dS/m (Spinach) others can stand up to 16 dS/m (date palm).

### Crop water response functions

In this section, we introduce the water response functions that will be used for the production assessment of the date palm plantation.

#### Land suitability-dependent water response function

The distinctive feature of the water response function is that it takes into account that new areas may have less suitable soil and hence lower yields. Five soil suitability classes are distinguished: the first class has no restrictions, while subsequent classes have increasing limitations, expressed as downgrading factors.

Given the water deliveries per ha, crop composition, costs per ha and salinity levels, we calculate net revenue, taking into account the suitability classes and the corresponding downgrading factors, as follows

$$\rho_{\text{d}} = \sum\limits_{k} {\left( {\sum\limits_{z} {A_{kz} r_{z} } \bar{y}_{k} p_{k}^{\text{f}} - A_{k} \left( {\sum\limits_{j} {u_{jk} } - p^{\text{w}} w_{k} } \right)} \right)}$$

where $\rho_{\text{d}}$ is the total net revenue from cropping in district *d*, per calendar year, in USD; $A_{kz}^{{}}$ denotes crop area; $A_{k}$ in soil suitability class $z$, in ha; $r_{z}$ is the downgrading factors for the yields, with $r_{1} = 1$; $\bar{y}_{k}$ is the yield of crop $k$ in the highest suitability class (*z* = 1), in ton/ha; $p_{k}^{\text{f}}$ is the farm gate price of crop $k$, in USD/kg; $u_{jk}$ is the cost of input $j$ (other than water) for crop $k$, in USD/ha; $p^{\text{w}}$ is the water price paid by crop farmers, in USD/m^3^; $w_{k}$ is the use of irrigation water by crop $k$, in m^3^/ha.

**Fig. 3:**
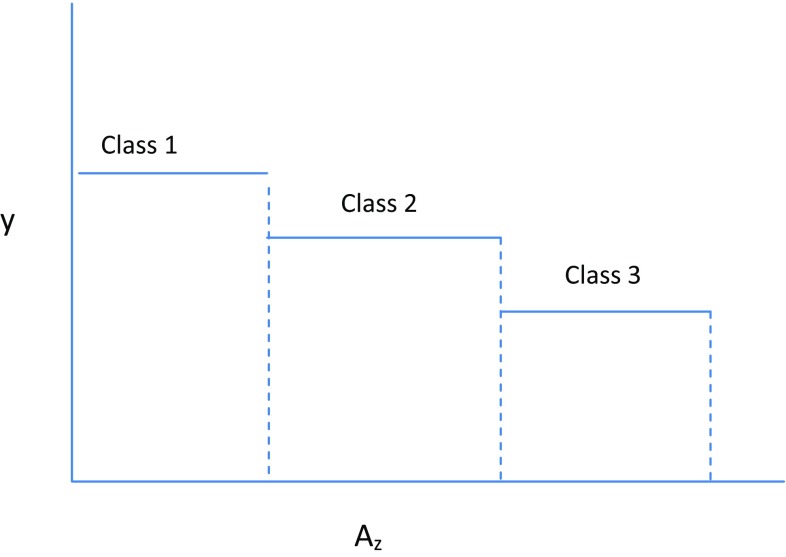
Linear dependency between yield (*y*) and area (*A*_*z*_) for three land suitability classes (*z*)

Graphically, the relationship between suitability classes and yield of a specific crop is depicted in Fig. 3.

#### Changing salinity levels

**Fig. 4:**
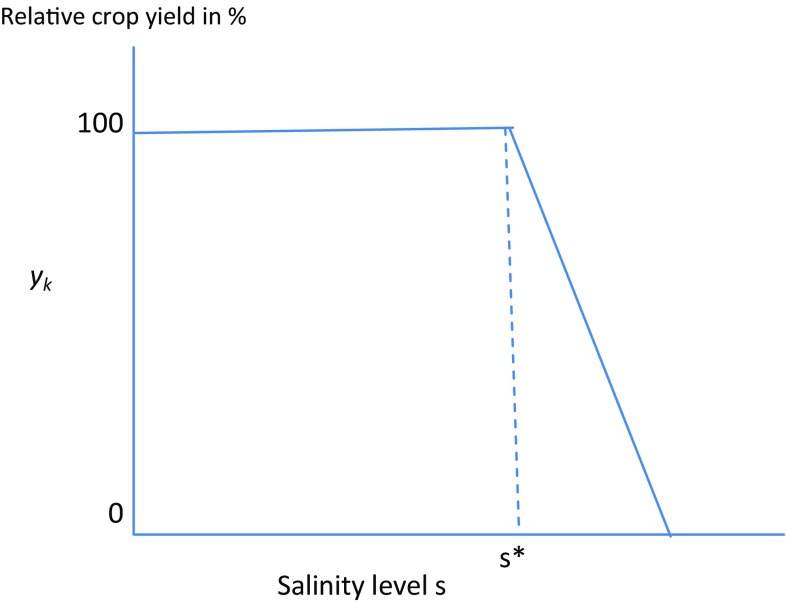
Relation between salinity and relative crop yield. s* indicates tolerable salinity level

We also account for the variations in water quality by calculating the crop-specific relationships between salinity and date palm yield according to the relationship:

$$y_{k}^{{}} = \gamma_{k}^{{}} (s)\bar{y}_{k}^{{}}$$

where $\gamma_{k} (s)$ gives the relative yield change in crop *k* under salinity level *s* with respect to the maximum yield ($\bar{y}_{k}$) under tolerable salinity levels; $\gamma_{k} (s)$ is calculated by the piece-wise linear equation:

$$\gamma_{k} (s) = \hbox{min} \left( {1,\hbox{max} \left( {0,K_{k}^{\text{s}} s + h_{k} } \right)} \right)$$

where $K_{k}^{\text{s}}$ and $h_{k}$ are date palm-specific parameters with values of − 5.46 and 114.75, respectively. Graphically, the equation is represented in Fig. 4.

Application of the response function requires a protocol that prescribes how the increased availability of irrigation water determines the surface of the newly cultivated areas of date palm and which type of land is taken into cultivation. For this, we assume that:

1. increase in water deliveries induces an increase in irrigated area under date palm cultivation such that new areas receive the same deliveries per unit as existing harvested date palm areas;
2. most suitable classes of land for date palm cultivation are used first.

In this way, crop response functions can be evaluated and used as input for the cost–benefit analysis.

### Cost–benefit analysis

Cost–benefit analysis (CBA) is a systematic approach for calculating and comparing benefits and costs of a project in monetary terms (Turner et al. 2004). Comparison of benefits and costs at different time scales requires a discounting factor that expresses future costs or benefits at today’s equivalent value. There is in principle no agreement which discount rate should be used and its selection is often controversial because it influences whether net benefits are positive or negative. A range of discount rates will be used in simulation.

We report on two indicators, the net present value (NPV) and the benefit–cost ratio (BCR). NPV determines whether projects should go ahead or not. A positive NPV indicates that project revenues compensate for investments and current costs and hence that a profit can be made. The NPV equation is:

$$npv = \sum\limits_{t = 0}^{T} {\frac{{b_{t} - c_{t} }}{{\left( {1 + r} \right)^{t} }}}$$

where *T* is the time horizon, *b* benefits, *c* costs and *r* the discount rate. BCR divides total discounted benefits by total discounted costs to estimate benefits for every monetary unit of investment.

## Results

### Water balances

In this section, we discuss in detail the water flows of the rainfall and balances of root zone and aquifer.

#### Surface layer

**Fig. 5:**
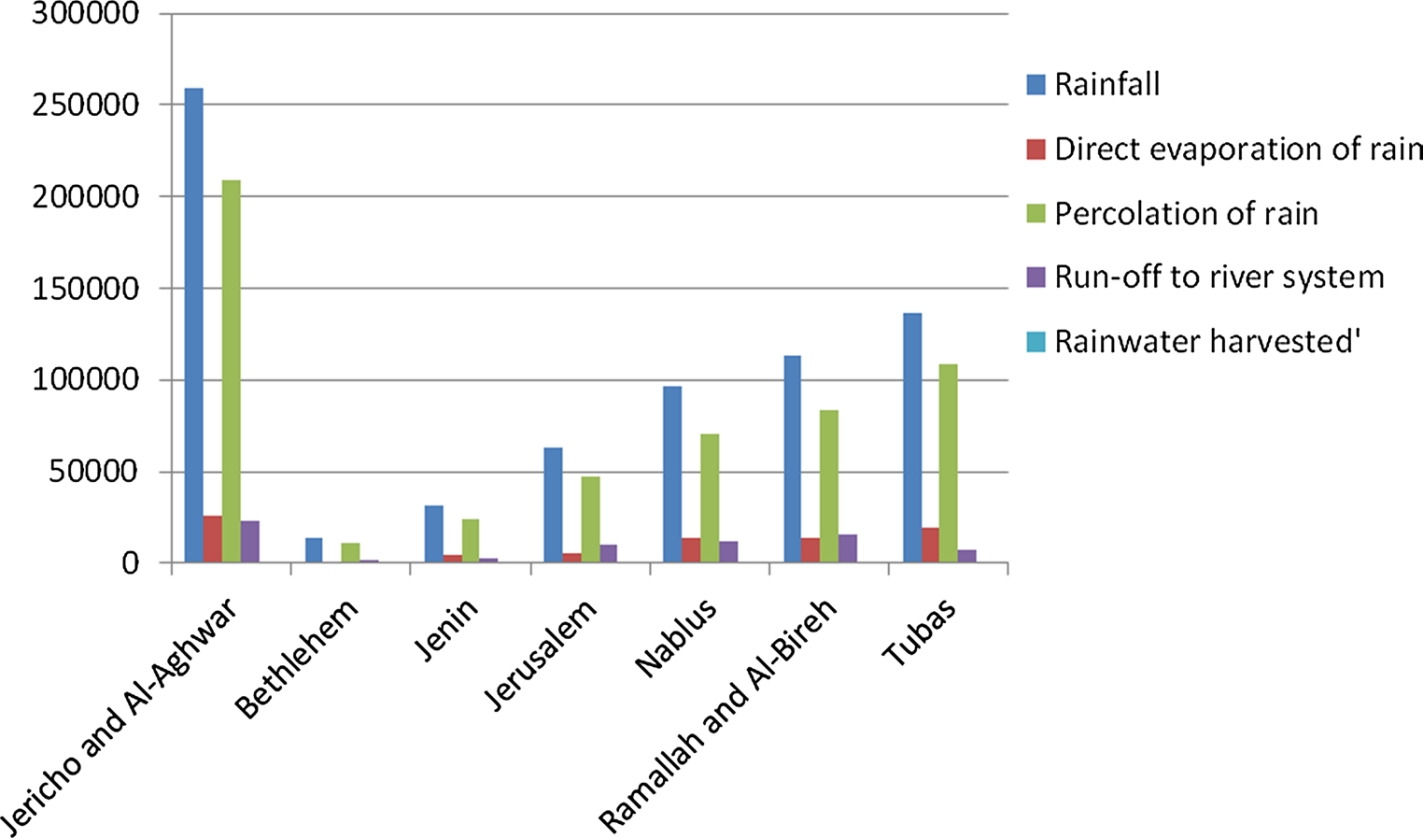
Annual rainwater balance (1000 m^3^) in the West Bank (*Source* Van Veen et al. 2016)

Approximately 75% of the precipitation falls in the period from December to February. Spatially, rainfall distribution follows prevailing topography where within a short horizontal distance of 45 km altitude it varies from 1004 m in the western mountains where rainfall of 550 mm/year is reported to − 410 m below sea level at the Dead Sea shoreline with an average rainfall of 150 mm/year. We discuss the hydrological setting in the West Bank using baseline information on balances of rainfall, root zone and aquifer, from the Concerted Sharing project (van Veen et al. 2016). Figure 5 shows the rainfall balance of the West Bank districts in the JRB. The total volume of rainwater is about 714 MCM (million cubic metres). The highest volume, 217 MCM, is received in Jericho-Al Ghoor District due to the large catchment area of about 725 km^2^, with average rainfall of 375 mm; Ramallah-Al Bireh district with a catchment area of 283 km^2^ contributes with 113 MCM/year. Approximately 23% of the rain percolates to the aquifer and 55% to the root zone, 12% evaporates, resulting in small runoff rates of 10%. In general, surface runoff is not used due to restrictions for dam construction and collection in ponds in the C-areas (areas under Israeli control). Only during the last few years, one small earth dam and a few collection ponds have been constructed with total storage capacity of about 1 MCM. All surface water within JRB flows eastwards and discharges into the Jordan River. Surface runoff infiltrates in the karstified underground characterized by fractured carbonate rocks of the mountain aquifer (Shadeed et al. 2007).

#### Root zone

Rain percolation is the main contributor to the root zone (Table 3), with smaller portions from irrigation sources: groundwater (8%), leakage from surface waters (6%) and springs (4%). Evapotranspiration from crops (17%) and non-agricultural land (52%) are major outgoing flows jointly with the percolation to deeper layers (24%). Leakage water percolating to deeper layers and evaporating in the air is minor outgoing water flows. Net-stocks for the root zone are negative except for Jericho-Al Ghoor and Jerusalem districts.

**Table 3:** Annual root zone balances for West Bank districts in the JRB: incoming and outgoing flows (1000 m^3^)

	Jericho and Al-Aghwar	Bethlehem	Jenin	Jerusalem	Nablus	Ramallah-Al Bireh	Tubas
Percolation rainwater	209,281	11,421	23,912	47,069	70,178	83,650	109,016
Leakage surface	14,243	816	775	8372	3714	9775	2615
Groundwater irrigation	36,624	37	1897	608	4787	1101	11,886
Surface water irrigation	1410	11	62	31	188	74	1485
Waste water irrigation	183	0	0	0	0	0	0
Spring water irrigation	9700	0	0	6600	8300	0	5200
Total incoming water	271,441	12,284	26,646	62,680	87,168	94,600	130,202
Crop evapotranspiration	27,359	1000	11,585	3783	24,509	8675	42,399
Evaporation non-agric	143,432	7788	11,120	34,541	41,816	60,185	61,813
Evaporation leakage water	11,047	119	487	2454	3212	1353	4032
Percolation deeper layers	58,187	3778	7153	16,714	21,123	27,787	28,778
Leakages deeper layers	10,971	91	469	2285	3026	1213	3976
Total outgoing water	250,995	12,776	30,814	59,778	93,686	99,212	140,998
Net water stock increase	20,446	− 492	− 4168	2902	− 6518	− 4611	− 10,796

#### Aquifer

Direct percolation from the root zone contributes 88% to the aquifer balance (Table 4), with delayed leakages providing for another 12%. Pumping (50%), springs from the aquifer (30%) and lateral flows to the river system (15%) account for the outgoing flows. Stock changes are relatively small, fluctuating mostly between one and six MCM except for Ramallah-Al Bireh district where a positive 27 MCM is reported. We can conclude that the root zone balance is predominantly negative and that possibilities for further groundwater exploitation to expand cultivated area are limited, not only for restrictions imposed by Israel, but also because inflow and outgoing flow are much in equilibrium and further extraction would jeopardize its sustainability.

**Table 4:** Annual aquifer balances for West Bank districts in the JRB: incoming and outgoing flows (1000 m^3^)

	Jericho and Al-Aghwar	Bethlehem	Jenin	Jerusalem	Nablus	Ramallah-Al Bireh	Tubas
Percolate. from root zone	58,187	3778	7153	16,714	21,123	27,787	28,778
Leak. from root zone	10,971	91	469	2285	3026	1213	3976
Net lateral inflows	19,000	0	0	0	0	0	0
Total incoming water	88,158	3869	7622	18,999	24,149	28,999	32,754
Groundwater pumping	43,051	409	2846	1181	10,633	1960	14,565
Spring water	9700	0	0	11,000	8300	0	11,300
Lateral outflow to river	14,161	0	0	0	0	0	5839
Total outgoing water	66,912	409	2846	12,181	18,933	1960	31,704
Net stock	21,246	3460	4776	6818	5216	27,039	1050

At the level of the groundwater basin, we observe that replenishment of the carbonate mountain aquifer sufficiently compensates withdrawals, while replenishment of the shallow aquifer system in the Jordan Valley is limited due to high evaporation and low rainfall rates (less than 250 mm/year). Groundwater flow regimes of both aquifers are eastwards and to the southeast (Dead Sea) direction. From the upper part of the mountain aquifer system, natural springs are channelled for domestic use and irrigation while deep groundwater boreholes with depth range between 400 and 600 m mostly serve Israeli settlers. Water quality in this aquifer is excellent with electrical conductivity of less than 1.5 dS/m (Wolf and Hötzl 2011). The shallow aquifer system consists of alluvial deposits where lateral replenishment is limited by the few contact zones along the fault system. This aquifer is used mainly by Palestinians for agricultural activities. Overall, recharges are lower than abstraction causing declining water tables and increasing salinity levels reaching 4.5 dS/m (Wolf and Hötzl 2011).

#### Underutilized water sources

Several water sources remain unused. For example, the treated wastewater from Al Bereh and Jericho is 3 MCM, while potential wastewater from Nablus East has a potential annual discharge of 3 MCM. The annual captured flooding water is about 2 MCM. The most important source of water to sustain future investment in date palm tree cultivation is the Al Faksha spring group located at the north-western shoreline of the Dead Sea, about 12 km from Jericho city. Its discharge ranges between 50 and 80 MCM and drains directly to the Dead Sea. Salinity of this group ranges between 2 and 7 dS/m with an average of 6.11 dS/m. The transfer of this source to Jericho needs additional infrastructure (pipeline, pumping stations, electricity and reservoirs).

### Land balances

For the assessment of land suitability for date palm cultivation, we apply a rule-based procedure (e.g. FAO 2002) that uses the principle of Liebig’s law of the minimum to control the most limiting factor. This is implemented by defining the ‘very suitable’ class as a combination of a class lower or equal to 3 assigned for the attribute slope and all first classes for the remaining land and soil attributes. The ‘suitable’ class has a class lower or equal to 3 assigned to the attribute slope, class 1 for reference depth and AWC, while at least one of the other attributes has class 2. The ‘moderately suitable’ class has a class lower or equal to 3 for slope and class 1 for reference depths and AWC, but at least one of the other attributes has class 3. The ‘moderately unsuitable’ class has a class lower or equal to 3 for slope and class 2 for reference depths and AWC, while at least one of the other attributes has class 3. Unsuitable are areas where slope class > 3. We assume that the first class has no restrictions, while subsequent classes reduce yields realized in the first class by a factor of 0.8, 0.6, 0.4 and 0 for classes two, three, four and five, respectively.

**Fig. 6:**
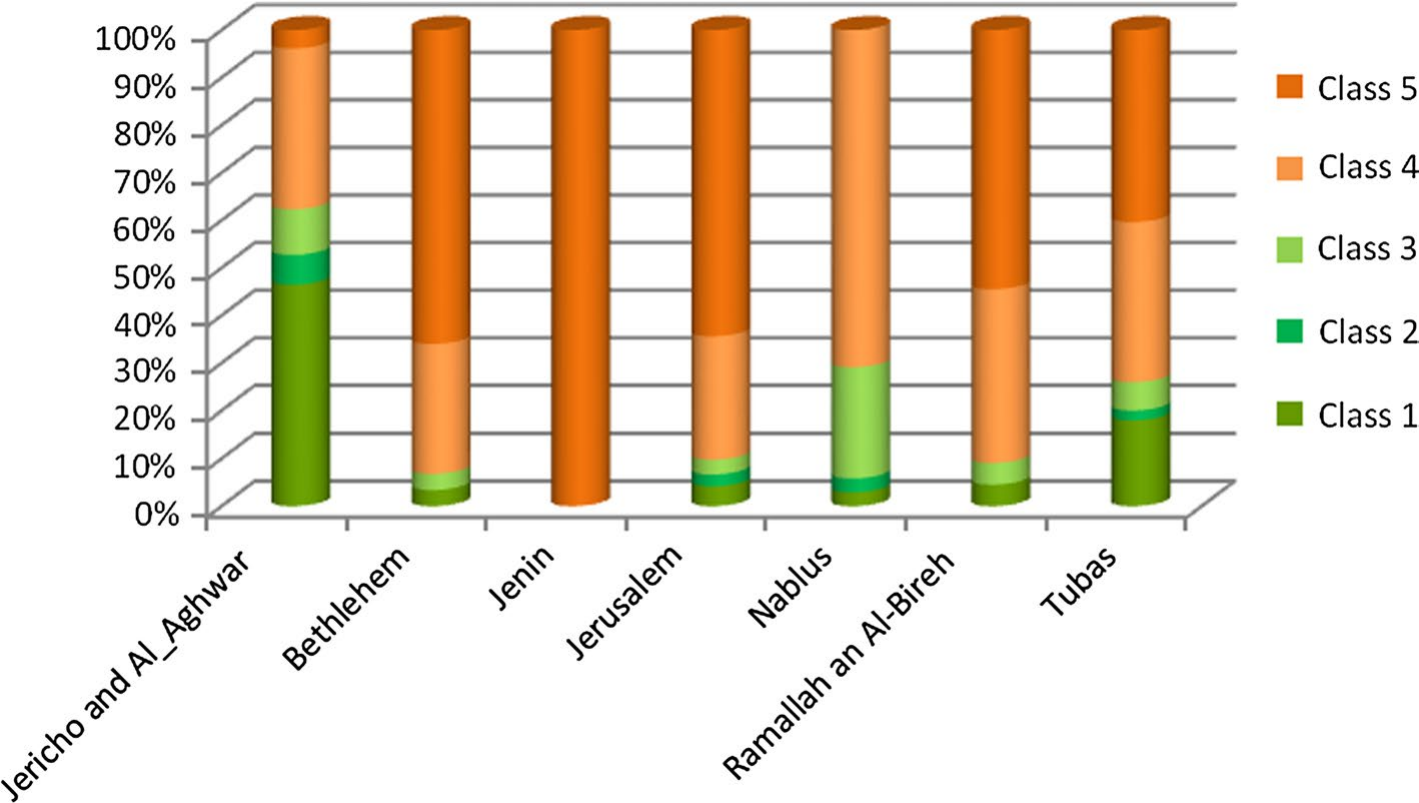
Share of land suitability class by district for date palm cultivation

Table 5 summarizes the area in ha for suitability classes by district in the JRB. Largest areas are found in Jericho-Al Ghoor and the Tubas Districts, followed by Nablus, Ramallah-Al Bireh and Jerusalem. Jenin and Bethlehem have little potential. Area shares of the suitability classes by district are shown in Fig. 6 and confirm the high potential of date palm cultivation in Jericho-Al Ghoor and to a lesser extent in Tubas. This is confirmed by the existing cropping areas of date palm, which are highly concentrated in the Jericho-Al Ghoor district, see Table 6.

**Table 5:** Land availability (in ha) by land suitability class for date palm cultivation by district in the JRB part of the West Bank

District/suitability class	Very suitable	Suitable	Moderately suitable	Moderately—unsuitable	Unsuitable
Jericho-Al Ghoor	33,242	4470	6858	25,134	2767
Bethlehem	124	0	124	990	2403
Jenin	0	0	0	0	7499
Jerusalem	670	393	495	4121	10,265
Nablus	706	706	5650	17,181	0
Ramallah-Al Bireh	1289	0	1289	10,309	15,434
Tubas	6465	721	2140	12,078	14,488

**Table 6:** Current date palm cultivation in ha by district

District	Irrigated	Rainfed	Total cultivated
Jericho-Al Ghoor	741.5	33.8	775.2
Bethlehem	0.0	0.0	0.0
Jenin	0.1	0.0	0.1
Jerusalem	0.1	0.0	0.1
Nablus	0.2	0.6	0.8
Ramallah-Al Bireh	0.4	0.0	0.4
Tubas	14.0	2.7	16.6

#### Available land

Land is occupied by the currently cultivated crops (Fig. 2), and in order to assess the potential for area expansion we calculate land availability as follows. We assume that the best suitable land for date palm cultivation is also preferred by other crops. When the cultivated area fully occupies the best suitable area, the remaining cultivated area is allocated in the next to the best suitable class, and so forth. The resulting land balance indicating available land for date palm cultivation by suitability class is presented in Table 7. We infer that Jericho-Al Ghoor district is the best place to expand date palm cultivation. Areas in Tubas and Nablus seem only moderately suitable at best, and ground truthing is needed to verify whether our assumptions on preferred area occupation hold.

**Table 7:** Land availability for date palm cultivation in ha by district in the JRB part of the West Bank

District/suitability class	Very suitable	Suitable	Moderately suitable	Moderately—unsuitable	Unsuitable
Jericho-Al Ghoor	27,391	4470	6858	25,134	2767
Bethlehem	0	0	59	990	2403
Jenin	0	0	0	0	4848
Jerusalem	0	348	495	4121	10,265
Nablus	0	0	2223	17,181	0
Ramallah-Al Bireh	0	0	1043	10,309	15,434
Tubas	0	0	0	10,180	14,488

### Cost–benefit analysis

Table 8 shows one-time costs in USD/ha and lifetime for date palm cultivation. Table 9 shows the yearly recurring costs for the husbandry of the date palms. Table 10, finally, shows the benefits of the date palms that start bearing fruits after 4 years and produce for eight consecutive years shoots that can be used for replanting. To minimize the impact of the initial years, when there is no production but investment costs have to be made, we perform the cost–benefit analysis over a 40-year period, which includes the full life cycle of the date palm (30 years) and a full replanting scheme realized in the last 10 years.

**Table 8:** One-time costs and their lifetime for date palm cultivation

Costs	USD/ha	Lifetime
Seedlings	6112	na
Planting	1950	na
Water/labour/fertilizer	3900	na
Costs irrigation	170	10
Bags	1698	3
Plastic box	1698	5
Fence	2377	10

**Table 9:** Recurring costs for date palm cultivation

Year	USD/ha
1	3900
2	6500
3–30	7800

**Table 10:** Benefits of date palm cultivation over the years

Year	kg/tree	kg/ha (130 trees)	USD/ha	Shoots/tree	Shoots/ha	USD/ha
1	0	0	0	0	0	0
2	0	0	0	0	0	0
3	0	0	0	0	0	0
4	20	2600	7800	0	0	0
5	30	3900	11,700	0	0	0
6	40	5200	15,600	0	0	0
7	50	6500	19,500	0	0	0
8	60	7800	23,400	1	130	5200
9	70	9100	27,300	1	130	5200
10-30	80	10,400	31,200	1 (till 14 years of age	130(till 14 years of age	5200 (till 14 years of age

We compute that the 52 MCM that become available from the underutilized resources can supply an additional 3525 ha of land. Based on the land suitability and availability assessment, we divide the expanded area over Jericho-Al Ghoor (90%: 3173 ha), Nablus (5%; 176 ha) and Tubas (5%; 176 ha). Furthermore, we assume that in each district the newly cultivated areas are developed over a time period of 10 years, with equal expansion areas in each year. The last 10 years are used to replace the date palms with new trees. Concerning the discount rates, after consultation with counterparts we decided to take rates of 3, 5 and 10%, thereby covering realistic possibilities for agricultural projects in the West Bank. We perform sensitivity analysis of the NPV results for various discount rates and salinity levels.

We apply the land suitability-dependent and salinity sensitive water response functions to calculate the NPV and BCR of the date palm production under various salinity levels and discount rates for Jericho-Al Ghoor, Nablus and Tubas. The results are presented in Tables 11, 12 and 13, for Jericho-Al Ghoor, Nablus and Tubas, respectively.^4^ We see for Jericho-Al Ghoor that under optimum levels of salinity and discount rates the NPV amounts to 580 Million USD, which amounts to 4572 USD/ha/year; for every invested USD 1.8 USD is returned. Under average salinity conditions, NPV fluctuates between 77 and 372 million USD with revenues of 603 and 2930 USD/ha/year for discount rates of 3 and 10%, respectively. Under a higher salinity level (7 dS/cm), only discount rates of 3 and 5% give positive NPV’s, while highest salinity levels (10 dS/cm) only give negative NPV results and BCR lower than 1. We note that under current salinity levels the NPV remains positive in Jericho-Al Ghoor for all discount rates. This is not the case for Nablus and Tubas. In Nablus, we see that under average salinity conditions date palm cultivation is not profitable for the highest discount rate. Under the low salinity levels, the NPV fluctuates between 1 and 17 million USD with revenues of 152–2711 USD/ha/year, clearly reflecting the less suitable land and soil conditions when compared to Jericho. In Tubas, date palm cultivation is clearly no option, even when water of good quality would be available.

**Table 11:** Net present value of date palm cultivation in million USD for Jericho-Al Ghoor cost–benefit ratio under various discount rates (3, 5, 10%) and salinity levels (2.00, 6.19 (average), 7.00 and 10.00) and accounting for land suitability

Salinity (dS/m)	Discount rate (%)					
	3		5		10	
	NPV	BCR	NPV	BCR	NPV	BCR
2.00	580	1.79	382	1.75	141	1.57
6.19	372	1.51	239	1.47	77	1.31
7.00	107	1.15	59	1.12	− 2	0.99
10.00	− 228	0.69	− 169	0.67	− 100	0.60

**Table 12:** Net present value of date palm cultivation in million USD for Nablus District under various discount rates (3, 5, 10%) and salinity levels (2.00, 6.19 (average), 7.00 and 10.00) and accounting for land suitability

Salinity (dS/m)	Discount rate (%)					
	3		5		10	
	NPV	BCR	NPV	BCR	NPV	BCR
2.00	19	1.51	9	1.36	1	1.08
6.19	10	1.26	4	1.14	− 1	0.90
7.00	− 2	0.96	− 4	0.87	− 4	0.69
10.00	− 16	0.58	− 13	0.52	− 8	0.42

**Table 13:** Net present value of date palm cultivation in million USD for Tubas District under various discount rates (3, 5, 10%) and salinity levels (2.00, 6.19 (average), 7.00 and 10.00) and accounting for land suitability

Salinity (dS/m)	Discount rate (%)					
	3		5		10	
	NPV	BCR	NPV	BCR	NPV	BCR
2.00	− 9	0.77	− 7	0.75	− 4	0.67
6.19	− 13	0.65	− 10	0.63	− 6	0.56
7.00	− 19	0.49	− 14	0.48	− 8	0.42
10.00	− 27	0.30	− 19	0.29	− 10	0.25

## Conclusions

We conclude that future date palm cultivation under current conditions and assumed water deliveries can be a profitable undertaking in the Jericho-Al Ghoor District and suggest that public investments into connecting water infrastructure to the Al Faksha springs are warranted. The water transfer from the Al Faksha springs anyhow widens possibilities for agricultural development in general and, hence, does not only depend on the success of date palm cultivation.

Methodologically, this study shows how various aspects of the land and water quantity and quality can be represented and quantified in the crop response functions. This is an important improvement for the CBA that, conventionally, accepts land to be of a uniform quality and does not account for the prevailing geographical diversity of land qualities and water salinity levels.

Yet, this study makes a number of assumptions that require special attention. First, we assume that market prices respond inelastically to increased supply. This only holds approximately if the presumed partners in export markets remain interested and display a proportional increase in demand. Evidence from the literature review suggests that there is still room for extra production. Second, salinity usually oscillates between minimum and maximum levels and we did assume it stays above the minimum tolerable salinity levels. Under deteriorating climatic conditions, additional desalination processes would be required. Third, unexpected diseases (e.g. Bayoud disease, Black scorch disease) might affect productivity and would require additional phytosanitary measures. Hence, we recommend a gradual expansion of the area combined with consistent monitoring of external factors that influence the economic and biophysical feasibility of the date palm cultivation.

Despite these risks, prospects for date palm expansion seem promising are economically feasible and generate an important social benefit as approximately 0.7 person years/ha can be employed, which means that more than 2700 persons can enter remunerative employment on the West Bank. This consideration may prove to be a strong argument in the process of drafting a concrete development plan for date palm cultivation in the West Bank.

A follow-up of this study should concentrate on changes in hydrological balances that might be the result of this large-scale undertaking, using the multilayer description of the connected water flow in the Jordan River Basin. Such a study would indicate, inter alia, the effects on the groundwater reserves and reuse of irrigation water and provide a broader underpinning of agricultural development in the West Bank.

## Footnotes

1 From now on when we refer to date palm it is understood to be the Majhool Date Palm variety.

2 This spatially explicit data set was compiled under the Concerted Sharing Project (http://acwfs.vu.nl/en/research/themes). It relates natural and controlled water flows and water quality in the JRB to crop and livestock production, industry, municipalities and households at district level for two-weekly time steps in the year 2010.

3 For soils with a reference depth below 100 cm the AWC in the Harmonized World Soil database is given in mm.

4 Detailed calculations on benefits and costs over time and age of the date palm cultivation are available on request.
